# Gonadotropin Regulated Testicular RNA Helicase, Two Decades of Studies on Its Structure Function and Regulation From Its Discovery Opens a Window for Development of a Non-hormonal Oral Male Contraceptive

**DOI:** 10.3389/fendo.2019.00576

**Published:** 2019-08-29

**Authors:** Maria L. Dufau, Raghuveer Kavarthapu

**Affiliations:** Section on Molecular Endocrinology, Division of Developmental Biology, Eunice Kennedy Shriver National Institute of Child Health and Human Development, National Institutes of Health, Bethesda, MD, United States

**Keywords:** GRTH/DDX25, spermatogenesis, round spermatids, androgen, hormonal regulation

## Abstract

Gonadotropin Regulated Testicular Helicase (GRTH/DDX25) is member of the DEAD-box family of RNA helicases present in Leydig and germ cells. GRTH is the only family member regulated by hormones, luteinizing hormone, through androgen action. Male mice with knock-out of the GRTH gene are sterile, lack sperm with arrest at round spermatids. GRTH participates on the nuclear export and transport of specific mRNAs, the structural integrity of Chromatoid Bodies of round spermatids, where mRNAs are processed and stored, and in their transit to polyribosomes, where it may regulate translation of relevant genes. GRTH has a central role in the control of germ cell apoptosis and acts as negative regulator of miRNAs which regulate expression of genes involved in the progress of spermatogenesis. In Leydig cells, GRTH gene transcription is regulated by LH via autocrine actions of androgen/androgen receptor and has regulatory effects in steroidogenesis. In germ cells, androgen actions are indirect via receptors in Sertoli cells. Transgenic mice carrying GRTH 5′ flanking region-GFP permitted to discern regions in the gene which directs its expression upstream, in germ cells, and downstream in Leydig cells, and the androgen-regulated transcription at interstitial (autocrine), and germ cell (paracrine) compartments. Further evidence for paracrine actions of androgen/androgen receptor is their transcriptional induction of Germ Cell Nuclear Factor as requisite up-regulator of GRTH gene transcription in round spermatids, linking androgen action to two relevant germ cell genes essential for the progress of spermatogenesis. A missense mutation of R to H at amino acid 242 of GRTH found in 5.8% of a patient population with azoospermia causes loss of the cytoplasmic phospho-GRTH species with preservation of the non-phospho form in transfected cells. Mice with knock-in of the human mutation, lack sperm due to arrest at round spermatids. This model permits to discern the function of phospho-GRTH. The GRTH phospho-site resides at a Threonine structurally adjacent to the mutant site found in patients. Molecular modeling of this site elucidated the amino acids that form the GRTH/PKA interphase and provide the basis for drug design for use as male contraceptive.

## Discovery and Early Studies

Gonadotropin regulated testicular RNA helicase (GRTH) was discovered in this laboratory in 1999 (1) as a result of screening for LH/hCG responsive genes using differential displayed analysis of RNAs from cultured rat Leydig cells stimulated by high concentrations of gonadotropin. Our intent at the time was to search for a master switch to gain further insights on gonadotropin-induced desensitization of steroidogenic enzymes previously described in our laboratory (2–4). Following single-strand conformation polymorphism analysis, sequencing of the fragments, and verification by RNA protection a single up-regulated fragment that displayed no similarity to any gene of the DATA bank was obtained. Upon screening a rat Leydig cell library a sequence (1,629 bp) that contained an open reading frame encoding 369 amino acids was revealed (1). The *in vitro* transcribed/translated rat cDNA gave a protein of 43 kDa. A data base search indicated similarity of GRTH sequence with members of the DEAD-box family of RNA helicases. In analyzing further clones, we found a single base pair addition which resulted on an opening reading frame of GRTH that contained 357 additional base pair 5′ to the original cDNA ATG codon. The complete sequence thus contains 1,630 bp and an open reading frame of 483 amino acids (5). Although our study did not provide the key to the desensitization process it unveiled a major advance with the discovery of GRTH/DDX25, a testis specific RNA helicase a novel member of the DEAD-box family of RNA helicases. Although this helicase was found initially in Leydig cells soon it was realized that the major function of GRTH is exerted in the germinal epithelium at post-meiotic stages of spermatogenesis. GRTH to date is the only helicase known to be stimulated by hormones. Its transcription is stimulated by gonadotropin (hCG/LH) induced androgen (A) formation/action through stimulation of cell surface LH receptors in Leydig cells and by paracrine action of androgen through Sertoli cells in germ cells of the rat and mouse testis. During the last 20 years, studies in this laboratory have concentrated in understanding the role of GRTH in spermatogenesis and Leydig cell function, its regulation and role in male reproduction.

## Characterization of GRTH Enzymatic Activity and Its Localization in Leydig and Germ Cells

**Figure 1:**
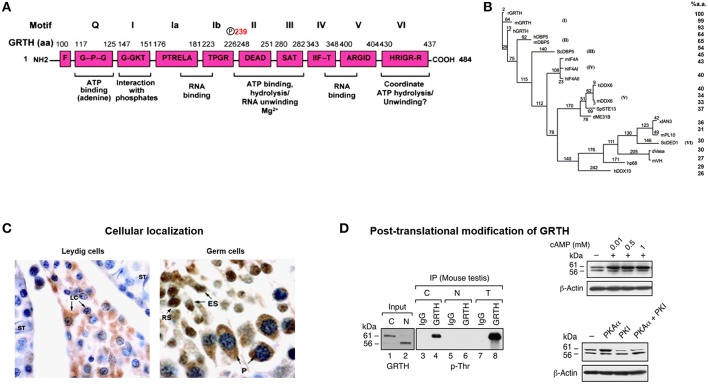
GRTH structure, cellular localization, and post-translational modification. **(A)** Schematic representation of the conserved amino acid motifs shared by GRTH with members of the DEAD-box family of proteins. Nine conserved motifs are indicated as (Q, I, Ia, Ib, II–VI). The amino acid position T239 showing phospho-modification in GRTH which is located outside the conserved domain. **(B)** Phylogenic analysis of members of DEAD-box family of RNA helicase. Maximum parsimony analysis of the alignment of 20 members of DEAD family was constructed using the neighbor-jointing method of PAUP 4.0. The mouse and human GRTH proteins display sequence similarity to the rat of 98.8 and 93.4%, respectively (7). **(C)** Cellular localization in mice testis of GRTH protein in the interstitial compartment and in different cell types in the rat seminiferous tubule (5). GRTH expression is noticed in Leydig cells (LC), pachytene spermatocytes (P), round spermatids (RS), and elongating spermatids (ES). **(D)** Western blot analysis (left) showing pull-down of GRTH protein from mice total testicular lysates (T) and purified cytoplasmic (C) or nuclear (N) fraction using GRTH specific antibody by immunoprecipitation. GRTH; p-Ser, p-Thr, and p-Tyr, phosphoantibodies were used to develop the blot. Right. Western blot showing effect of cAMP treatment on COS-1 cells overexpressing GRTH (above). Western blot showing increased on post-translational modification (phospho) of overexpressed GRTH protein species by co-transfected PKA catalytic subunit (pGRTH, 61 kDa and non-phospho GRTH, 56 kDa) prevented by PKA inhibitor (PKI) (below) in COS-1 cells (8).

GRTH/DDX25 was the designated trivial/scientific name assigned to this novel RNA helicase. It contains all 9 conserved motifs of the DEAD (Asp-Glu-Ala-Asp)-box RNA helicase family members (Q, I, Ia, Ib, II III, IV, V, VI) including those involved in RNA binding and ATP interaction (6). GRTH contains ATPase activity and ATP dependent RNA helicase activity (Figure 1A). It differs from other members of the family in possessing high intrinsic ATPase activity in absence of RNA, but as other family members its activity is highly enhanced in presence of mRNA, synthetic Pol A and DNA while Poly U, total RNA and tRNA, had only minor effect. The ATPase activity either intrinsic or stimulated has an essential Mg^2^ requirement. GRTH unwinds messages bidirectionally, experimentally double strands of RNA/RNA, and RNA/DNA 3′ and 5′ duplexes were unwound by GRTH-GST in presence of ATP. GRTH was cloned from the rat Leydig cell, mouse and human testis cDNA libraries (1). Besides the conserved motifs characteristic of the DEAD-box family GRTH has low amino acid sequence similarity with most other members of the family. Its unique N- and C-terminal sequences and overall structure presumably confers its multiple specialized biochemical functions. GRTH mRNA and protein are predominantly expressed in the male gonad and minor levels of the transcripts are expressed in brain, hypothalamus, pituitary and other tissues and stable cell lines in culture including those of rat anterior pituitary and hypothalamic neurons (1, 7). GRTH displays 93–98% amino acid sequence similarity among rat, mouse and human species. It has 64% amino acid identity with DBP5/DDX19 (human/mouse/yeast) which is ubiquitously expressed, associates with nuclear pore complex (9–11) and is required for RNA poly (A) export function (Figure 1B). It displays considerably less similarity (41–42%) to initiation factors of the translation complex, eIF4AI,II/DDX2A,B (12) and only 32–34% to other germ cells DEAD-box RNA helicase proteins including, An3 which is expressed throughout oogenesis, embryogenesis and adult life and colocalizes with nucleoli in *Xenopus laevis* oocytes (13), Ded1p/PL10 (*S. cerevisiae*/mouse) require for translation (14) and Vasa/mVH/DDX4, a DEAD-box helicase family member essential for male germ cell development and oogenesis in Drosophila (15). It is also found in the mammalian male germ cell lineage where in its absence spermatogenesis is blocked at the first meiotic cell division (16).

## GRTH Species Its Generation and Post-transcriptional Modifications and Their Cellular Localization

Multiple protein GRTH species were generated through usage of three alternative translation initiation codons from a single transcript of 1.6 kilobases. Testicular germ cells preferentially utilize +1 ATG which closely match the consensus Kozak sequence and yield major proteins of 61/56 kDa. The Leydig cells utilize the 2nd ATG at +343 position resulting in the expression of 48/43 kDa forms. Only in germ cells weak utilization of the 3rd ATG codon at+ 568 producing a 33 kDa protein species was observed (5). ATG codons are utilized in cell specific manner and at least one is hormonal dependent (gonadotropin/cAMP/androgen), since there is switch from 1st to 2nd ATG utilization upon *in vitro* hCG stimulation which was reversed by androgen antagonist Flutamide (5). This was observed in round spermatids not in pachytene spermatocytes. The 2nd ATG is utilized in Leydig cells where endogenous androgen production is abundant and in round spermatids under exogenous hCG treatment which increases androgen levels/actions in the seminiferous tubule (Sertoli cells). Thus, we conclude that utilization of the 2nd ATG is dependent on androgen induced factor(s). This concept is reinforced by more recent findings which demonstrated that the GRTH in round spermatids is transcriptional up-regulated by androgen while pachytene spermatocytes showed no response (17).

The 56 kDa GRTH is the non-phosphorylated form present in the nucleus of germ cells which transports specific nascent messages to the cytoplasm via Chromosomal Maintenance 1 (CRM1) pathway (Figure 1D). It is also present in small quantities in the cytoplasm and at this site the function of these species has not been explored. The leucine-rich region at the N-terminal comprising amino acids 61–74, was identified as the nuclear export signal which participates in CRM1-dependent nuclear export pathway of relevant mRNAs to cytosolic sites. Moreover, deletion analysis has localized the GRTH nuclear localization sequence to amino acid positions 100–114, 5′ adjacent to the second ATG codon. The 61 kDa GRTH phospho-species is present solely in the cytoplasm of germ cells [Figure 1D; (8)]. This GRTH form by association with relevant messages prevents their degradation and presumably participates in the transport of mRNA to Chromatoid Bodies (CB) of round spermatids for storage prior to translation at specific times during spermatogenesis. The 61 kDa phospho-form also participates in the transport of messages to actively translating polyribosomes where it is believed to engage in the translational regulation of germ cell specific genes involved in the progress of spermatogenesis. Phosphorylation of GRTH is induced by cAMP dependent Protein Kinase A Cα at Threonine 239 [Figure 1D; (8, 18)]. This single site resides in the core region, not within any of the conserved motifs of the family. Its PKA site sequence TKIR is a non-canonical motif which is more common in the testis than in other tissue (19). In the case of 48/43 kDa species it is proposed to represent the phospho-/non-phospho species, respectively, however their specific function need to be established. Both lower molecular forms (48/43 kDa) lack the 5′ sequences where the known nuclear import and export localization sequences for the 61/56 kDa are present, and its intrinsic localization sequences if present remain to be defined. With our present knowledge the 43 kDa form is not expected to reside in the nucleus or engaged in transport of messages, but perhaps its actions are confined to translational process of mRNAs.

## Cellular Developmental Expression of GRTH in the Testis

Initial localization of GRTH by *in situ* hybridization in the rat revealed the presence of GRTH transcripts in interstitial and germ cells of the testis. Immuno-labeling was observed within the seminiferous tubules of adult rats in meiotic cells, pachytene spermatocytes and haploid round and elongated spermatids. In contrast, germ cells within the basal compartment (spermatogonia and preleptotene spermatocytes) and Sertoli cells were negative. In pubertal cells only pachytene spermatocytes were positive (1). GRTH mRNA expression was not observed in testis of immature rats, 8–12 days old, while positive signals were observed in tubules and Leydig cells of pubertal animals (ages 23 and 26 days) and in the adult testis. Although there are currently no studies on GRTH expression in neonatal animals, at times when testosterone and LH levels are known to be elevated, it is likely that GRTH expression in neonatal Leydig cells could be temporarily elevated. Northern blot analysis detected a single transcript of 1.6 kb in Leydig and germ cells of the testis and small abundance of transcripts in the hypothalamus, pituitary, and brain but was not found in ovary or other tissues examined including uterus, liver, kidney, heart and adrenal. Also, equivalent size transcripts were detected in the adult human and mouse testis (1). Early *in vitro* studies using recombinant GRTH-GST showed 100–200% increased translation of luciferase RNA template over control indicating the potential role of GRTH in translation. The developmental and stage specific expression of GRTH mRNA pointed to a Gonadotropin/androgen dependence and a participation of this helicase in spermatogenesis. *In vivo* stimulation by hCG of GRTH mRNA in Leydig cells were also observed *in vitro* and similar effects were found upon stimulation of cells with cAMP (8-bromo cAMP) *in vitro*, and an equivalent stimulation of transcripts was induced upon incubation with dihydrotestosterone. Increases induced by hCG and cAMP were prevented by inhibitor of steroidogenic enzymes (cholesterol side chain cleavage, 3β-hydroxysteroid oxidoreductase and 17α-hydroxylase/17-20 desmolase) which abolished the androgen production induced by hCG/cAMP indicating its androgen dependence in Leydig cells (8).

The development of an affinity purified GRTH peptide antibody (amino acid positions 465–467) identified GRTH as a developmentally regulated protein in Leydig and germ cells. Initial immunohistochemistry studies in the rat showed GRTH immunoreactive protein in the interstitial cells of the testis (8). In the seminiferous tubules both pachytene spermatocytes and round spermatids expressed GRTH protein and the intensity of staining varied at different stages of the spermatogenic cycle (Figure 1C). Staining in round spermatids attained maximal levels at stages VIII and IX and were reduced at later stages X-XIII of elongated spermatids. In pachytene spermatocytes GRTH protein levels were generally lower than in round spermatids gradually increasing from stages II to IX, and like round spermatids reached peak levels at stages VIII and IX declining thereafter at stages X through XIII. Intense protein staining was observed in stage XIV in spermatocytes entering the metaphase of meiotic division (there are 14 stages of the spermatogenic cycle in the rat and 12 stages in the mouse). In the mouse, peak levels of GRTH protein labeling is observed in round spermatids, pachytene spermatocytes, and elongated spermatids at stages VIII, IX, and X, respectively, and at stage XII is found in spermatocytes entering the metaphase of meiosis. GRTH protein was not found in myoid and Sertoli cells. Combined use of immunocytochemistry studies and western blot analysis demonstrated that GRTH is a developmentally regulated protein in Leydig and germ cells of the rat and mouse testis (8).

## Gene Structure, Cell Specific Expression, and Mechanisms of Regulation by Androgen in Leydig and Germ Cells

**Figure 2:**
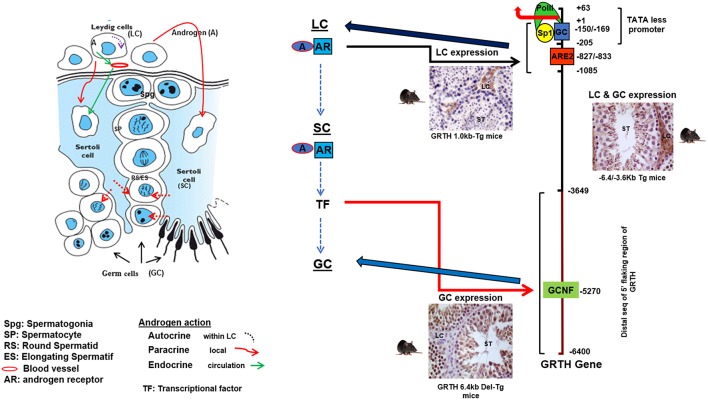
Androgen (A) regulated GRTH expression in cell compartments. Diagram elucidating the 5′-flanking sequence of GRTH gene that directs its cell-specific expression in testicular cells and direct/indirect actions of androgen on GRTH transcription at cellular compartments. A produced from LCs binds to AR and subsequently interacts with ARE2 (−828/−833) to direct GRTH gene expression in LCs. Paracrine activation (yet to be identify) by androgen from LC induces A/AR-responsive gene(s) expression in Sertoli cells. These in turn may activate downstream targets that directly or indirectly induce GRTH transcription through GCNF binding to 5′ elements located in the distal (−3,600/−6,450 bp) 5′-flanking region of the gene (17, 21, 22).

The GRTH/DDX25 gene resides in chromosomes 11q24 in human, 8q25 in rat and 9A3-A5 in mouse. The 20 KB mouse gene contains 12 coding exons, all but one of the conserved motifs which is shared between two exons 10 and 11 (motif V), reside within single exons and identical genomic organization was identified of the human GRTH gene. The GRTH gene promoter is TATA-less contains GC sequences, initiator elements, and multiple TSSs. The promoter resides within −205/+63 bp of the gene. The basal transcriptional activity is driven by essential Sp1/Sp3 binding sequences at −169/−150 within the promoter (20). GRTH is regulated by LH through Androgen/Androgen Receptor (AR) directly at the transcriptional level in Leydig cells with impact solely in gonadotropin stimulated steroidogenesis and testosterone production *in vivo* and *in vitro* with no impact in basal conditions. In germ cells however, the action of A is indirect through AR in Sertoli cells and its expression is both cell and stage specific. Sertoli cells are the nursing cells of germ cells with established cellular and molecular communications, thus A actions presumably proceed in a paracrine fashion through signaling events. Generation in our laboratory of transgenic mice models carrying GRTH 5′ flanking regions with GFP as reporter provided *in vivo* systems that permitted differential elucidation of regions that contain sequences in the GRTH gene that directs expression to different cellular compartments, upstream sequences (−6.4/−3.6 kb linked to its promoter −205 to+63-GFP) in germ cells and downstream (−1,085/+63 GFP) in Leydig cells (Figure 2). Androgen produced in Leydig cells under the pulsatile LH stimulus from the pituitary gland, stimulates transcription of the GRTH gene in an autocrine manner through its AR association with a non-canonical androgen response element half-site at −827/−822 (5′-TGTCC-3′). This occurs through an association of A/AR bound to its cognate DNA site with member(s) of the preinitiation complex, TFIIB and Pol II via a short-range chromosomal loop revealed by CHIP3C assays. Association of SRC-1 and Med-1 co-activator to A/AR, though not require for looping are essential for AR, TFIIB, and Pol II recruitment to the complex and GRTH transcriptional activation (21). Other studies demonstrated that in transgenic mice the −6.4 kb/+63 GFP transgene directed GRTH expression to both Leydig cells and germ cells (Figure 2). Androgen regulation of GRTH transcription *in vivo* and *in vitro* in the germ cell compartment is only confined to round spermatids. The expression of GRTH by androgen is regulated by androgen/AR acting in Sertoli cell which in a paracrine fashion regulate GRTH transcription through a yet to be identified signal(s) relayed to germ cells. This is accomplished by the participation of the transcription factor GCNF (Germ Cell Nuclear Factor) whose expression is regulated by androgen in round spermatids. In these cells GCNF associates to a consensus half-site (−5,270/5,252 nt) of the GRTH gene and promotes its cell specific regulated transcription/expression of this helicase. Moreover, GRTH has been found to be associated with GCNF mRNA and to have an inhibitory effect on GCNF message stability which demonstrate an autocrine regulation of GCNF by GRTH at the post-transcriptional level (22).

## Lessons Learnt on the Functions of GRTH From the GRTH-Targeted Null Mice

### Phenotype, Functional, and Structural Alterations

**Figure 3:**
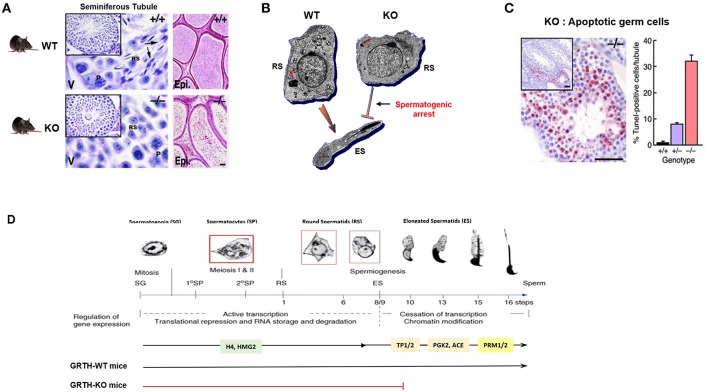
GRTH-KO mice model reveal the role of GRTH in the completion of spermatogenesis. **(A)** H&E staining of WT and KO mice testis showing seminiferous tubules and epididymis. Presence of elongated spermatids only in WT mice is indicated by arrows. P. pachytene spermatocytes; RS, round spermatocytes. Epididymis lumens of WT mice contains mature sperm while in KO mice lumens are filled with degenerating germ cells (23). **(B)** EM images of round spermatid from WT and GRTH-KO mice and elongated spermatid of WT mice. Absence of GRTH in KO mice causes arrest of spermiogenesis at step 8 of round spermatids and failure to elongate (23). CB of KO mice was highly condensed, greatly reduced in size and lacking the typical nuage texture (red arrow) as compared with wild type CB (red arrow). **(C)** TUNEL IHC staining of testis section from GRTH-KO mice show marked apoptosis in germ cells compared to WT mice. Quantitative evaluation of apoptosis (right), mean ± SE of apoptotic cells per tubule [10 tubules were assessed for each group (23)]. **(D)** Diagram of mice spermatogenesis showing spermatogonia (SG, 2n) followed by first meiotic (I) division resulting in the formation of primary (1°) and secondary spermatocytes (SP). Secondary SP through meiosis generate haploid round spermatids (RS) entering the differentiation process of spermiogenesis (16 steps in mouse) to produce elongating spermatids (ES) and spermatozoa/mature sperm. Germ cells expressing GRTH (spermatocytes and round spermatids) are boxed in red. The regulation of gene expression during the developmental process is governed in a precise temporal sequence: an initial active transcription phase with translational repression is followed by cessation of transcription associated with chromatin modification. Chromatin structure in spermatogenic cells changes during development from mitotic spermatogonia into meiotic SP and post-meiotic haploid spermatids. During spermatid elongation, histones are removed and replaced by TP1 and TP2 and subsequently by Prm1 and Prm2. GRTH- dependent expression of proteins is highlighted including H4 (testis specified histone), HMG2, TP2/TP1, testicular ACE, PGK2, Prm1/Prm2. RS development in the WT showing progression to mature sperm. In GRTH-KO, there is a spermatogenic arrest observed at step 8 of RS (7).

GRTH knock-out mice (homozygous) are sterile due to a blockade of spermatogenesis at step 8 of round spermatids resulting in complete lack of elongated spermatids and sperm and solely degenerating spermatids were present in the lumen of the epididymis (Figures 3A,D). These mice exhibit normal sexual behavior and have normal circulating levels of gonadotropins and testosterone. This excluded androgen deficiency as the cause of the spermatogenic arrest, despite the existence of EM abnormalities in the Leydig cells (23). These included marked reduction of lipid droplets, swollen mitochondria and lack of typical normal cristae structure while the circulating basal androgen levels were found to be normal in GRTH^−/−^ mice. However, subsequent studies revealed considerable testosterone increase upon stimulation of Leydig cells with high doses of hCG *in vivo* and *in vitro* in null mice when compared to WT. Changes in Leydig cell structure and function are in concert with marked accumulation of cholesterol in the inner mitochondrial membrane and an increase in protein expression of genes involved in cholesterol synthesis and transport including HMGCR, Srebp2, and StAR (24). In contrast, heterozygous mice are fertile. Females homozygous have normal fertility consistent with GRTH expression solely in the male gonad.

Of major note was the highly condensed chromatoid bodies (CB) of markedly reduced size with lack of the usual “nuage” appearance at all steps of round spermatids in GRTH^−/−^ when compared to GRTH ^+/+^ and GRTH ^+/−^ mice (Figure 3B). These changes in the CB of null mice are consistent with their lack of GRTH dependent nuclear-cytoplasmic transport of messages concern with the progress of spermatogenesis (5, 8). The CB is a non-membranous filamentous cytoplasmic body which resides in the cytoplasm adjacent to the nucleus of round spermatids and serve as repository of long-lived mRNAs associated as mRNPs waiting for translation during spermatogenesis. CB contains members of the small interfering RNA pathway like MIWI, argonaute protein, Risc/Dicer endonuclease, decapping enzyme, and mi/si/pi RNAs that participate in the RNA-mediated silencing/degradation (25). CB also contain MVH/DDX4, a mouse homolog of drosophila VASA, which is commonly used as a germ cell marker (15, 26) and proposed to participate in the small RNA interfering pathway to regulate RNA processing (25). MVH has an essential role in the MILI, MIWI, and MIWI2 dependent piRNA processing pathway (27). It is of note that GRTH is highly abundant in the CB (28, 29). In elongating spermatids, the CB moves to a caudal site at the base of the flagellum, where it subsequently undergoes fragmentation and disappears from the cytoplasm (30). There is limited information about the specific mechanisms of the CB function during spermatid elongation. In late pachytene spermatocytes a series of small granules which associated with the nuclear envelope and reside between small vesicles are believed to be precursor of CBs (31). More recent studies have indicated that piRNAs originated in pachytene spermatocytes from non-transposon intergenic regions cause massive abrogation of cellular programs in elongated spermatids in preparation of sperm production (32). CB are believed to possess functional similarities to P-bodies and stress granules which contain aggregates of translationally repressed mRNPs associated with the translation repression and mRNA decay machinery in early development in neurons (33, 34). P-bodies contain members of mRNA decapping machinery, including the decapping enzymes and its activators. Stress granules share some components in common with P-bodies, typically contain translation initiation factors eIF4E, eIF4G, eIF4A, eIF4B, poly-A binding protein, eIF3, eIF2, and the 40S ribosomal subunit (25). Further, the CB also resembles the recently described TIS associated granules and the interconnections proposed between TIS granules and ER (TIGER domain structure) could apply to the CB (35). In germ cells/round spermatids, mRNAs are transported from nucleus to the cytoplasm by GRTH via CRM1 pathway where messages are temporarily stored and translationally repressed in the CB awaiting translation during spermiogenesis and where can also undergo degradation. Disruption of GRTH gene did not show changes on steady state mRNA levels of chromatin remodeling gene transcripts and other of relevance to the progress of spermatogenesis including Tp1, Tp2, tACE. This was observed on the background of reduced cytoplasmic levels, which was attributed to their impaired nuclear export. However, their protein was absent in the null mice indicating the post-transcriptional function of GRTH (Table 1) (7). Because in KO mice the arrest occurs prior to the elongation where chromatin remodeling proteins are normally expressed in elongated spermatids, it is difficult to assess the relevance of GRTH in the posttranscriptional event but given their association with GRTH in transport and presumably at cytosolic sites it is strongly indicated the prevalent post-transcriptional function of this helicase (8).

**Table 1:** Summary - GRTH, a multi-functional protein regulating several cellular events.

**Cellular function/Event**	**Role of GRTH**
Apoptosis	GRTH prevents germ cell apoptosis by regulating expression of several genes pro-apoptotic/anti-apoptotic genes (Cas3, Cas8, Cas9, PARP, Bid, Bad, Bak, p53, Bcl2, Bcl-xL, IκBα/β, TRADD) in the Caspase, NFκB, and TNFα/TNF-R1 mediated pathways.
RNA Transport	Participates in nuclear export of germ cell specific mRNAs (Tp2, Prm2, PGK2, tACE) from nucleus to cytoplasmic sites during spermatogenesis
RNA Processing	GRTH as a helicase protein helps in RNA unwinding. It selectively binds to germ cell specific mRNAs and polyribosomes for active translation process during spermatogenesis
RNA Degradation	GRTH prevents degradation of Tp2, Prm2, and Tssk6 mRNA's essential for spermiogenesis
miRNA Regulation	GRTH plays an important role in the negative regulation of testis specific miRNAs (miR469, miR34c, miR470) and other miRNAs like Let7 family members through regulating their biogenesis via Drosha/DGCR8 microprocessor complex
Spermatogenesis	GRTH is essential for spermatid development and completion of spermatogenesis (round spermatid to elongating spermatid). Maintains the integrity of chromatoid body which stores germ cell specific RNAs during spermatogenesis.

### GRTH in microRNA Regulation

A set of primary microRNAs were up-regulated in round spermatids of GRTH KO mice. These include testis specific miR-469, testis preferred miR-34c and miR-470 and others such let-7 family members (let-7a/d, b and e-g) and miR203 (Table 1). Also, the enzyme complex (Drosha-DGCR8) required to process the Pri-miRNA transcripts to Pre-miRNAs in the nucleus is upregulated in the KO mice This occurs prior to their transport via exportin 5 to the cytoplasm and final processing by Dicer dependent pathways to mature miRNA with residence in the CB. Our studies have identified TP2 and Prm2 as target genes for miR-469. Through binding to the coding regions of the mRNAs of these chromatin remodelers miR-469 represses their protein expression. GRTH has an essential role in the negative regulation of a subset of miRNAs through maintaining their biogenesis via Drosha/DGCR8 microprocessor complex to generate mature miR 469 and others could play a role during spermatogenesis. Control of the temporal progression of spermatogenesis via miR-469 inhibitory action on TP2/Prm2 mRNA at the CB site could be essential for their timely expression for chromatin compaction in spermatids and the progress of spermatogenesis (36).

### Role in Apoptosis

In GRTH null mice, major apoptosis was predominant at stage XII of spermatogenesis and confined to spermatocytes entering the metaphase of meiosis, well before the arrest point (step 8 of round spermatids) and subsequently magnified at the various steps of round spermatids (23). This indicated that at the early stage, the development of germ cells were compromised and progression from a reduced number of cells followed. Spermatogenesis progression to haploid steps of round spermatids in the GRTH^−/−^ mice where apoptosis was so prevalent in pachytene spermatocytes (30% cells/tubule), indicated the participation of yet to be defined compensatory mechanism(s) during these important developmental stages of spermatogenesis (Figure 3C). These could include activation of survival mechanisms, noted by the increase of DNA repair proteins (Rad 51 and Dmc1) required for maintaining chromosomal integrity in GRTH null mice and the possible participation of other helicases such as DDX3 and DBP5 in the progression from the remaining viable meiotic cells to haploid steps of round spermatids until the arrest point at step 8. The enhanced apoptosis in the GRTH null mice is undoubtedly related to the absence of GRTH protein since apoptotic cells in spermatocytes were only reduced to 8% per tubule in GRTH^+/−^ mice and <1% in wild type mice. Comparative studies in pachytene spermatocytes of GRTH null mice vs. Wild type revealed that Pro- and anti-apoptotic factors are regulated by GRTH. Significant increased levels of pro-apoptotic factors Bid, Bak. Bad, Smac and p53 was observed in GRTH null mice while levels of anti-apoptotic proteins including Bcl2, Bcl-xL, phospho-Bad, and HSP10 were significantly reduced. Also, major reduction of PKA(c), Erk1/2 and pErk1/2, enzymes known to phosphorylate Bad (anti-apoptotic) was found in the GRTH null mice indicating the general effect of this helicase on the mitochondria directed apoptotic route (37). The lack of GRTH could reduce mitochondria integrity through the increases in membrane bound proapoptotic proteins that promote release of cytochrome C with significant effects on Caspase Signal Pathways. These included significant increases in active cleaved products of caspases 9 and 3 and PARP recognize to induce DNA fragmentation (Table 1). GRTH through its association with caspase 3 mRNA has a significant role in its stability through increased degradation, consequently marked increases in the half-life of the transcript was observed in the null mice. GRTH has effect on NFκB-mediated anti-apoptotic pathway. Increased levels of IκBα and reduction of its phosphorylation which cause sequestration of NFκB dimers in the cytoplasm was observed in the KO mice. This promotes association of IκBα/β with NFκB and prevents its nuclear translocation which is required for transcriptional activation of antiapoptotic genes. Moreover, GRTH regulates the TNFα/TNF-R1 mediated pathway and caspase 8 mediated events by regulating the levels of TRADD expression. GRTH associated with some apoptotic factors (Bad, Bac, Smac, p53) and anti-apoptotic factors (Bcl-2, Bcl-xl, HSp10). It also associates with caspases 3, 8, and 9, PARP, TRADD, and IκB and nuclear regulators of NFκB- p300 and HDAC1 (37). The prevalence of apoptotic or antiapoptotic pathways could result from association of corresponding transcripts with GRTH which in turn cause silencing/degradation or alter translation and transport events.

## Role of Phospho-GRTH in Spermatogenesis

**Figure 4:**
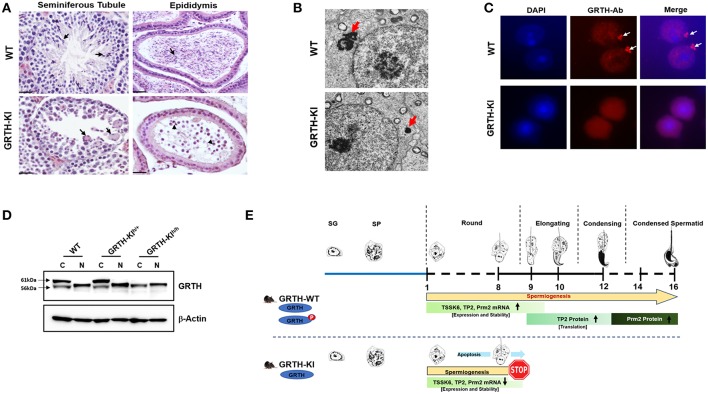
GRTH-KI and essential role of phospho-GRTH in round spermatid during spermiogenesis. **(A)** H&E staining of WT and GRTH-KI mice testis showing seminiferous tubules and epididymis. In WT mice seminiferous tubules arrows indicate presence of elongated spermatids. GRTH-KI show degenerating multinucleated giant cells indicated by arrows. Epididymis of WT mice is filled with mature sperm (indicated in arrows) while in KI mice lack sperm but the lumens contain degenerating germ cells (indicated in arrowheads). **(B)** EM sections testis showing marked size reduction in chromatoid body (CB) in round spermatids (red arrows) of GRTH-KI mice compared to WT mice. **(C)** Immunofluorescence staining of GRTH protein (Red) in the nucleus and at cytoplasmic sites, in chromatoid bodies (CB) of round spermatids of WT mice (39). Arrows indicate CB. GRTH-KI mice lack GRTH signal in the CB. Nuclear staining using DAPI is shown in blue. **(D)** Western blot showing non-phospho GRTH expression in WT, heterozygous and homozygous KI mice while pGRTH expression was totally absent in homozygous KI mice compared to WT mice and heterozygous KI mice (39). **(E)** Schematic diagram showing progression of mice spermatogenesis from spermatogonia to round spermatids which undergo different steps (total 16) of development to give rise to a condensed sperm. In WT mice during the process of spermiogenesis in round spermatids we observed stable expression of Tssk6, Tp2, and Prm2 mRNAs and phospho-GRTH plays an important role in the stability of these germ cells specific mRNAs until ready for translation in later steps during elongation of spermatids. During spermatid elongation transition proteins are replaced by protamines1/2 and the chromatin becomes more condensed. While in GRTH-KI mice due to lack of phospho-GRTH the stability of above-mentioned germ cells specific mRNAs is hampered resulting in their reduced mRNA levels and degradation. In GRTH-KI mice Tp2 and Prm2 proteins are absent due to failure of round spermatids to elongate in step 8 of spermiogenesis (39).

Our early studies revealed that a missense heterozygous mutation of R249 to H of GRTH found in 5.8% of Japanese patients with non-obstructive azoospermia and 1% of normal controls, when expressed in COS1 cells, causes loss of the 61 kDa cytoplasmic phospho-GRTH species with preservation of the nuclear 56 kDa non-phospho form (38). From this initial finding we could conclude that the mutation observed in these patients was not the cause of azoospermia, as judge by our observation in null mice where only homozygous null mice were infertile (38). However, the finding provided an avenue to elucidate the function of phospho-GRTH in spermatogenesis. Recently we generated a humanized GRTH knock-in (KI) mouse with the R242 to H mutation observed in the patients (39). Homozygous mice are sterile with marked reduction in the size of the testis which lack sperm (Figure 4A) with arrest at step 8 of round spermatids and complete loss of the cytosolic phospho-GRTH species (61 kDa) with preservation of the non-phospho form of GRTH (Figure 4D). In KI mice the androgen and gonadotropin levels were comparable to the Wild Type and the mating behavior is normal. In contrast, heterozygous mice are fertile. The non-phospho form is predominant in the nucleus and small quantities are also present in the cytoplasm of the KI mice. Thus, in KI mice the nuclear transport functions were preserved while the cytoplasmic functions including shuttling of messages, storage in the CBs and translational events which require phospho-GRTH are absent. Consequently, a marked reduction of the chromatoid body of round spermatids was revealed (Figure 4B). KI mice lack phospho-GRTH protein in CBs (Figure 4C). Germ cell apoptosis was observed in pachytene spermatocytes and round spermatids until the arrest point. In contrast to KO, KI mice revealed no changes in miRNA biosynthesis which indicated involvement of non-phospho rather than phospho-GRTH most likely as a transcriptional regulator of members of the microprocessor complex Drosha DCGR affecting primiRNAs formation (36). In KI mice we found loss of chromatin remodeling and related proteins including TP2, PRM2, and TSSK6 and also a significant decrease of their mRNAs and half-lives, indicating that their association with phospho-GRTH in the cytoplasm protect mRNAs from degradation (Figure 4E). Moreover, we demonstrated that mRNAs concerned in spermatogenesis bound phospho-GRTH and their association to actively translating polyribosomes. These and related transcripts were found down-regulated at polysomal sites in KO mice. Ingenuity analysis predicted association of pGRTH bound messages at polysomal sites of round spermatids with the ubiquitin-proteosome-heat shock protein network and the NFκB/TP53/TGFB1 signaling (40). In early studies we demonstrated that GRTH protein bind to the 3′ UTR region of mRNA (41). In very recent functional studies, we have shown that phospho-GRTH has an important role in the translation of Tp2 through binding of its 3′ untranslated regions (39).

## The GRTH Phospho-site and Its Connection With pKAα Catalytic -as the Initial Step for Development of a Non-hormonal Male Contraceptive

The identification of the GRTH phospho-site at threonine (T239) (18) structurally adjacent to the mutant site (R 242H) found in patients (38) provided a frame for modeling of relevant amino-acids that form the GRTH pocket/PKAα catalytic interphase. Molecular modeling based on the RecA domain 1 of DDX19 (18, 42, 43) elucidated the relevant amino acids that formed the pocket, solvent accessibility and H-bonding, which upon disruption caused reduction or abolition of the phospho-GRTH form proven essential for completion of spermatogenesis. These include in addition of the core residues at T239 and R242 amino acids E165, K240, and D237. It is relevant to note that the deleterious effects on GRTH phosphorylaton caused by the mutations are not engendered from changes of PKAα-catalytic binding affinity but in the arrangement of the pocket for efficient catalytic activity of the kinase (18). Blocking the phosphorylation of GRTH at T239 through perturbation of the pocket should provide an effective selective and specific oral non-hormonal male contraceptive.

## Author Contributions

MD and RK conceived the concept of this review and wrote the manuscript.

### Conflict of Interest Statement

The authors declare that the research was conducted in the absence of any commercial or financial relationships that could be construed as a potential conflict of interest.
