# Corrigendum: Gonadotropin Regulated Testicular RNA Helicase, Two Decades of Studies on Its Structure Function and Regulation From Its Discovery Opens a Window for Development of a Non-hormonal Oral Male Contraceptive

**DOI:** 10.3389/fendo.2019.00710

**Published:** 2019-10-18

**Authors:** Maria L. Dufau, Raghuveer Kavarthapu

**Affiliations:** Section on Molecular Endocrinology, Division of Developmental Biology, Eunice Kennedy Shriver National Institute of Child Health and Human Development, National Institutes of Health, Bethesda, MD, United States

**Keywords:** GRTH/DDX25, spermatogenesis, round spermatids, androgen, hormonal regulation

In the original article, there was an error in the ***Title***, first instance of the word “Regulation” should have been “Regulated.”

A correction has been made to ***Title***:

“Gonadotropin Regulated Testicular RNA Helicase, Two Decades of Studies on Its Structure Function and Regulation From Its Discovery Opens a Window for Development of a Non-hormonal Oral Male Contraceptive”

The authors apologize for this error and state that this does not change the scientific conclusions of the article in any way. The original article has been updated.

